# Improving flavor of strong fragrant rapeseed oils by supplementing commercial peptides and sugars

**DOI:** 10.1016/j.fochx.2024.101985

**Published:** 2024-11-08

**Authors:** Zi-Xiong Zhou, Yu-Jun Chen, Ming-Ming Sheng, Feng-Jie Cui, Chen Chen, Jian-Cheng Shi, Xue-Quan Shu, Zhi-Wei Chen

**Affiliations:** a School of Food and Biological Engineering, Jiangsu University, Zhenjiang 212013, PR China; b Jiangsu Hefeng Grain and Oil Industry Co., Ltd., Yancheng 212002, PR China; c Yancheng Hengxi Biotechnology Co., Ltd., Yancheng 224100, PR China; d Jiangsu Jiafeng Grain and Oil Industry Co., Ltd., Yancheng 224100, PR China; e Inspection and Testing Center of Dafeng, Yancheng 224100, PR China

**Keywords:** Fragrant rapeseed oils, Flavor improvement, Peptides, Reducing sugars, Maillard reaction

## Abstract

The strong fragrant rapeseed oils (SFROs) attract an increasing consumers' preference due to their strong flavor and attractive appearance. With the accumulated knowledge of flavor formation pathways during producing SFROs, the present study proposed a novel method to enhance SFRO's flavor by directly adding the reducing sugars (glucose and xylose) and commercial peptides during roasting process. Results indicated that supplementation of 3 % rapeseed peptide, 1 % glucose and 0.5 % of xylose gave an attractive color of SFRO with highest red value of 6.5, highest sensory score, enhanced nutty and roasted fragrance and distinguishing sweet flavor. The contents of typical volatile compounds such as pyrazines and furans in R-SFRO also showed the highest levels, proving that addition of the commercial peptide and reducing sugars as the Maillard reaction substrates could significantly enhance the flavor. Additionally, the proposed method showed potency for the large-scale application due to the simple steps, and low-cost input.

## Highlights

• An economic and efficient method was proposed to enhance the flavor of SFROs.
• The optimal treatment was addition of 3.0 % rapeseed peptide (R-SFRO) and sugars.
• R-SFRO showed the attractive color with highest red value of 6.5.
• R-SFRO had the highest flavor score and contents of Maillard reaction products.
• Percentages of low MW peptides/amino acids are the key factor to improve the SFROs' fragrance.

## 1 Introduction

Rapeseed oil is a widely consumed edible oil for cooking, frying, or baking with high affordability and versatility (Zhang et al., 2024). Approximately 32.84 million tons of rapeseed oils were produced in 2023, which ranked the third position after those of palm oil and soybean oil (77.57 million metric tons and 59.03 million metric tons, respectively) (USDA National Agricultural Statistics, 2023). Generally, the increase of rapeseed oil market mainly comes from its interesting nutritional credits such as the high contents of functional compositions including unsaturated fatty acids (oleic acid, linoleic acid and linolenic acid), vitamin E and sterols (Wang, Fan, & Li, 2023; Wang, Li and Wu, 2023). The unique flavor is another key attribute to attract the increasing market (Shen et al., 2023). Currently, at least two kinds of commercial rapeseed oils including cold- and hot-pressed rapeseed oils are on sale (Siger et al., 2017). Among them, cold-pressed oil produced by conditioning rapeseeds at 100 °C, and pressing at 50 °C, is more preferred by the European consumers, while the Chinese market shows an increasing interest on the hot-pressed rapeseed oils due to the strong flavor and attractive appearance from the high-temperature roasting (160–200 °C) and pressing (100–150 °C) processes.

Chinese market classifies the rapeseed oil products to strong-, mellow-, and light- fragrant types (SFRO, MFRO, and LFRO, respectively) based on their aroma intensity, which mainly comes from the refining steps including degumming, deacidification, decolorization, and/or deodorization steps (Chen et al., 2024). Among them, the strong fragrant rapeseed oil (SFRO) is produced by high-temperature (140–200 °C) roasting and screw-hot pressing (>100 °C) of rapeseeds, and refined with hydration degumming and/or dewatering with diatomite (T/CCOA 1–2019), which maximally retain the characteristic flavor, chlorophyll, and pigments offering the a dark-brown color with typical fragrant flavor and highest contents of nutrients including vitamin E and sterols. The SFROs have occupied 30 % oil market in China with an annual consumption of about 150 million tons (Kraljić et al., 2018; Zhou et al., 2019). From these points, improvement of the fragrant flavor intensity and nutrient contents of SFROs have received an increasing interest to significantly accelerate the enhancement of the quality, consumers' preference, and marketing targets (Tan et al., 2022; Tan et al., 2024).

The rapeseed oils' flavor mainly originates from three pathways including glucosinolate degradation, lipid thermal oxidation and Maillard reaction, producing over 300 volatile compounds such as nitriles sulfur (*S*)-containing compounds, pyrazines, aldehydes, furans, and nitriles (Zhang et al., 2022). The existing results proved that the microwave, ultrasound, and infrared roasting pretreatment of rapeseeds and processing parameters to enhance the glucosinolate degradation and lipid thermal oxidation significantly affected the flavor intensity and nutritional quality of SFROs. For example, Zhang et al. found that 700-W microwave pretreatment on rapeseeds significantly increased the amounts of the volatile compounds from glucosinolate degradation, and improved the pungent, smoky and roasted tastes (Zhang et al., 2024; Zhang, Fu, et al., 2023). Increase of substrate supply of the Maillard reaction such as carbonyl compounds and amino compounds also is an efficient method to enhance the flavor. Han et al. found that supplying the fructose and the alkaline protease-treated rapeseed meals resulted in the high total tocopherol content (597.78 ppm) and sterol content (790.00 ppm) and increased sweeter nutty/toasted flavor with a reasonable fatty acid composition and benzo(*a*)pyrene content (1.43 ppb) (Han et al., 2023). Our previous work also presented an enzymatic method to enhance the flavor properties of SFROs by neutral protease and α-amylase-treatments of rapeseeds (NRO + ARO). By supplying the reducing sugars and amino acids from α-amylase and neutral protease hydrolysis, the produced SFRO presented a strong nutty and rich roasted fragrance and distinguishing sweet flavor with the highest sensory score (Tan et al., 2024).

However, the microwave, ultrasound, and infrared roasting, and/or enzymatic pretreatments of rapeseeds have various limitations for industrial application such as high-cost input of proteases/ amylases and equipment, and tedious steps to select suitable enzymes, and specific reactors and drying ovens, which would significantly increase the overall production cost of SFROs (Han et al., 2023; Zhang et al., 2024; Zhang, Zhen, et al., 2023). Hence, on the basis of our previous results (Tan et al., 2024), the present study aimed to develop an economical method to enhance the Maillard reaction by supplying the substrates of commercial rapeseed, soybean, corn and peanut peptides and reducing sugars (glucose and xylose) to increase the flavor intensity of SFROs with the acceptable overall physical/chemical/ nutritional qualities. The outputs would provide an economic and low-cost proposal to develop the series of SFROs products with enhanced flavors.

## 2 Materials and methods

### 2.1 Materials and chemicals

The commercial rapeseed, soybean and corn peptides were purchased from Henan Gaobao Industrial Co., Ltd. Peanut peptide was purchased from Henan Zhongchen Biotechnology Co., Ltd. Their detailed physical and chemical properties including protein and sugar contents, molecular weight distributions of peptides, free amino acid compositions/contents were assayed and listed in Table 1. The rapeseed seeds were provided by Jiangsu Hefeng Grain and Oil Industry Co., Ltd. The vitamin E (*α*-, *γ*-, *δ*- tocopherols), sterols (5α-cholestanol, β-sitosterol, brassicastero and campsterol) and coenzyme Q_10_ were obtained from Sigma Chemical Co. Ltd. (Sigma, St. Louis, MO, USA) with the purity of over 98 %. 2-Octanol (GC grade) with the purity of over 99.5 % was purchased from Maclin Biochemical Technology Co., Ltd. Glucose, xylose, n-hexane, methanol and ethanol were obtained from Sinopharm Co., Ltd. (Sinopharm, Shanghai, China). Chromatograph-grade n-hexane, methanol and ethanol are purchased from Sigma (Sigma Chemical Co., St. MO, USA). Free amino acid standards and fatty acid methyl ester standards (GC grade) with the purity of over 99.5 % were obtained from ANPEL (ANPEL, Shanghai, China).

**Table 1:** Physical/chemical properties of commercial peptide samples.

Peptide types	Rapeseed	Soybean	Corn	Peaunt
Moisture/%	3.46 ± 0.25^a^	4.2 ± 0.82^a^	3.25 ± 0.34^a^	4.36 ± 0.88^a^
Reducing sugar(g/kg)	0.00	0.00	0.00	0.00
Protein contents(g/kg)	885.20 ± 12.25^a^	808.20 ± 10.64^a^	530.39 ± 6.62^a^	500.96 ± 4.38^a^

Molecular weight range (%)				
>10,000	0.05	0.04	0.08	67.92
10,000–5000	0.85	1.38	0.14	5.40
5000–3000	0.25	1.49	0.28	1.90
3000–2000	0.78	2.10	0.37	0.85
2000–1000	1.25	6.69	2.26	0.95
1000–500	9.88	18.64	10.26	0.77
500–180	56.40	53.72	62.16	5.45
<180	30.54	15.94	24.44	16.76

Amino acid(mg/kg)				
Asn	112.0 ± 1.6^a^	56.5 ± 0.8^c^	67.2 ± 1.1^b^	43.2 ± 0.4^d^
Glu	196.0 ± 1.7^b^	202.8 ± 1.9^a^	112.8 ± 1.2^d^	132.5 ± 1.5^c^
Ser	39.7 ± 0.5^a^	38.2 ± 0.4^b^	21.5 ± 0.3^c^	21.0 ± 0.2^c^
His	32.7 ± 0.5^a^	19.9 ± 0.2^b^	18.1 ± 0.2^c^	18.6 ± 0.6^c^
Gly	34.7 ± 0.3^a^	20.6 ± 0.2^c^	27.8 ± 0.8^b^	15.8 ± 0.3^d^
Thr	44.8 ± 0.5^a^	38.4 ± 0.3^b^	19.3 ± 0.2^c^	18.2 ± 0.8^d^
Arg	72.5 ± 0.6^a^	24.3 ± 0.2^d^	62.8 ± 0.5^c^	63.2 ± 0.6^b^
Ala	35.4 ± 0.3^a^	76.3 ± 0.3^a^	20.2 ± 0.3^a^	18.6 ± 0.3^a^
Tyr	24.2 ± 0.3^b^	34.7 ± 0.3^a^	18.7 ± 0.2^c^	16.7 ± 0.5^d^
Asp	2.5 ± 0.1^b^	3.9 ± 0.1^a^	2.7 ± 0.2^b^	2.1 ± 0.1^c^
Val	38.9 ± 0.3^a^	35.8 ± 0.3^b^	21.4 ± 0.5^d^	22.4 ± 0.3^c^
Met	12.1 ± 0.1^b^	23.4 ± 0.2^a^	5.4 ± 0.4^c^	12.2 ± 0.1^b^
Phe	43.1 ± 0.3^b^	46.9 ± 0.9^a^	27.6 ± 0.4^d^	32.5 ± 0.6^c^
Ile	41.2 ± 0.5^a^	36.2 ± 0.4^b^	19.6 ± 0.2^c^	18.6 ± 0.2^d^
Leu	62.80 ± 0.6^b^	130.90 ± 2.7^a^	33.4 ± 0.3^c^	25.4 ± 0.2^d^
Lys	61.6 ± 0.3^a^	13.4 ± 0.3^a^	18.9 ± 0.3^a^	5.8 ± 0.3^a^
Pro	30.3 ± 0.3^b^	62.4 ± 0.5^a^	18.4 ± 0.2^c^	13.6 ± 0.1^d^
Total	884.7 ± 4.3^a^	865.4 ± 6.8^b^	515.7 ± 5.8^c^	304.7 ± 3.8^d^

Note: Different letters indicate significant differences (*P* < 0.05). All experiments were performed in triplicates. The data were expressed as means ± standard deviations (*n* = 3). Results are presented as mean values ± SD; Noted: Asn, Glu, Ser, His, Gly, Thr, Arg, Ala, Tyr, Asp, Val, Met, Phe, Ile, Leu, Lys and Pro represented Asparagine, Glycine, Serine, Threonine, Arginine, Alanine, Tyrosine, Asparticacid, Valine, Methionine, Phenylalanine, Isoleucine, Leucine, Lysine and Proline.

### 2.2 Proximate analysis, free amino acid/sugar contents and molecular weight distribution of commercial peptides

The proximate compositions of commercial rapeseed, soybean, corn and peanut peptides, including moisture, crude protein, residual sugar and crude ash were determined according to the methods of AOAC (1990, 15th). The crude protein content was calculated using the nitrogen factor of 4.38.

The concentrations of free amino acids in the commercial peptides were determined using the method described by Liu et al. (Liu et al., 2020). Two grams of each commercial peptide sample was added into 20 mL of 50 g/L sulfosalicylic acid for 2 h. The supernatant was collected after 15-min centrifugation at 6000 ×*g*, mixed with 2 mL of hexane, and applied onto the Sykam S433D/S433 automatic amino acid analyzer (Sykam, Munich, Germany). Similarly, 2.0 g of each commercial peptide sample was mixed in the deionized water, and centrifuged at 4 °C 6000 ×*g*. The supernatant was analyzed with a HPLC system with an amino column (NH2P-50, 250 mm × 4.6 mm, Agilent Technologies Inc., Wilmington, USA) for determining the sugar compositions and their ratios with the column temperature of 30 °C, flow rate of 0.8 mL/min, and acetonitrile: water (75:25, *v*/v) as mobile phase.

The molecular weight distributions of the soluble peptides in commercial rapeseed, soybean, corn and peanut peptide samples were analyzed with the procedures provided in Chinese National Standard (GB/T 22492–2008). Briefly, the commercial peptide sample was added into 50 mL demonized water, stirring for 30 min through a 0.45-μm membrane filter. The filtrate was applied onto the gel permeation chromatography (GPC) using a TSK gel filtration column, 2000SWXL 300 mm × 7.8 mm (Tosoh Co., Tokyo, Japan) with the mobile phase of water/acetonitrile/trifluoroacetic acid (80/20/0.1, v/v/v). The standard proteins/peptides including insulin (5808 Da), bacitracin (1422 Da), tetrapeptide GGTA (451 Da), and tripeptide GGG (189 Da) were used to obtain the calibration curve (Y = -0.24× + 7.05, R^2^ = 0.98) further for calculating the molecular weight distributions.

### 2.3 Preparation of SFROs with supplementation of various commercial peptides and sugars

According to our previous results (Tan et al., 2024), the rapeseeds were mixed with 1 % (*w*/w) of glucose and 0.2 % (w/w) of xylose, and commercial peptides including rapeseed, soybean, corn and peanut peptide with the ratios of 1 %, 2 % and 3 % (w/w), respectively. Rapeseeds with no addition of commercial peptides and sugars were used as a control. The mixtures were roasted at 160 °C for 20 min using a continuous-cycle single-layer roaster (YJY-CY2, Yijiayi Machinery Equipment Co., Ltd., Hubei, China), and pressed at 80 °C with a screw press machine (YJY-150, Yijiayi Machinery Equipment, Hubei, China). The crude SFROs were degummed by adding 3 % of hot water and centrifuging at 6000 ×*g* for 15 min and stored at −4 °C for further analysis.

### 2.4 Physicochemical indexes

Acid value (AV) and peroxide value (PV) of all SFROs were assayed according to the methods Ca 5a-40 and Cd 8b-90 (AOCS, 2017). The AOCS Official Method Cc 13e-92, using a Lovibond Tintometer Color Scale at 70 °C, and AOCS Official Method Ca 2d-25 were used to determine the color, moisture and volatile matter content, respectively (AOCS 2017).

### 2.5 Analysis of fatty acid composition

According to the Al-Khalifa's method (Al-Khalifa, 1996), fifty micrograms of SFRO was added into 2 m L of ether and petroleum ether mix (1:1, *v*/v). After reacting with 2 mL of 3 M NaOH (in methanol) for 15 min, 5 mL deionized water was added and mixed vigorously. Fatty acid methyl ester (FAMEs) was obtained using a 0.22 μm polytetrafluoroethylene (PTFE) filter (Milicron, Inc., Bedford, MA, USA) in a volume of 1 mL, and analyzed with Shimadzu GC-2010 Pro gas chromatography (Shimadzu- Kyoto, Japan) equipped with an Agilent HP-88 column (100 m × 0.25 mm i.d., 0.20 μm film thickness) (Agilent Technologies Inc., Wilmington, USA). The GC–MS parameters were set as: carrier gas of nitrogen at the flow rate of 3.0 mL/min, the temperature of 160 °C for 5 min, increase to 220 °C at a rate of 1.8 °C/min and retain for 8 min. The injection volume was 1 μL. The fatty acid compositions in the SFRO samples were qualitatively and quantitatively obtained by comparing their retention time and peak areas with the FAMEs standards (Sigma Chemical Co., St. Louis, MO, USA).

### 2.6 Analysis of nutrients tocopherols, sterols and coenzyme Q_10_

The compositions and contents of the α-, γ- and δ-tocopherols were determined using HPLC referred to the method of Potočnik et al. (Potočnik et al., 2018). The SFRO sample was dissolved in n-hexane completely and applied onto the Shimadzu LC-20 A system (Shimadzu, Kyoto, Japan) equipped with a fluorescence detector and C18 column (250 mm × 4.6 mm, id, 5 μm film thickness; Agilent Technologies, Wilmington, USA). According to the retention time of α-, γ- and δ-tocopherol mix standard, the α-, γ- and δ-tocopherol contents were analyzed at λ_exc_292 nm and λ_em_325 nm with methanol-water (98:2, *v*/v) as the mobile phase at a flow rate of 0.8 mL/min (isostatic) absorbance was measured at λ_exc_292 nm and λ_em_325 nm and determined qualitatively and quantitatively.

The contents of sterols were determined using GC–MS methods described by Li et al. (Li et al., 2011). The oil sample mixed with 2 mg/mL 5α-Cholestane (Sigma Chemical Co., MO, USA) and 2 mol/L KOH at 75 °C for 1 h. After adding 5 mL of hexane and 4 mL of distilled water, the sample was silylated with 100 μL *N*, *O*-Bis(trimethylsilyl) trifluoroacetamide at 60 °C for 30 min, redissolved by 1 mL hexane. The temperature of DB-5 capillary column (0.25 μm, 0.25 mm × 30 m) (Agilent Technologies Inc., Wilmington, USA) was set as follows: held at 200 °C for 2.0 min, then increased to 300 °C at a rate of 10 °C/min and maintained at 300 °C for 15 min. The flow rate of the carrier gas (helium) was 1 mL/min, and the split ratio was 1:100 with the injection volume of 1 μL. The contents of sterols (mg/kg oil) in samples were quantitatively analyzed with internal standards.

The SFRO sample preparation for measuring the Coenzyme Q_10_ content was same as that for tocopherol determination (Lunetta et al., 2008). 10 μL SFRO/n-hexane solution was injected into the Shimadzu LC-20 A system (Shimadzu-Kyoto, Japan) with UV detector at λ_275_ nm and C18 column (250 mm × 4.6 mm, ids, 5 μm film thickness). The mobile phase was methanol-ethanol solution (10:90, *v*/v) at a flow rate of 1.0 mL/min. The Coenzyme Q_10_ content was calculated based on retention time and external standard method using Coenzyme Q_10_ standard (Sigma Chemical Co., St. Louis, MO, USA).

### 2.7 Sensory evaluation

The sensory evaluations including appearance, and flavor characteristics of prepared SFROs were conducted in the sensory laboratory according to ISO 8589-2007 (Sensory Analysis-General guidance for the design of test rooms) and GB/T 5525–2008 (Vegetable fats and oils-Method for identification of transparency, odor and flavor).

According to the established sensory descriptors, a sensory evaluation questionnaire was formed for rapeseed oil, and then a 16-assessor panelist including 7 males and 9 females at the ages of 23–35. These panelists from Jiangsu University and Jiangsu Hefeng Grain and Oil Industry Co., Ltd., were trained to differentiate the taste and aroma characteristics of SFROs including bitter, roasted, sweet flavor, musty, spicy, rapeseed flavor, nutty and soft. The assessors sniffed 6 samples each time and took a rest for 20 min for each 3 samples. 3 mL samples and heated to 40 ± 2 °C were placed in each covered cup and coded using a 3-bit random code. The samples were presented to the evaluators in a random order. The assessors were asked to select the aroma description smelled in the sample from the list of questionnaires descriptors and score it on the intensity range of 0 to 10 points from “not intense” to “very intense”. New aroma characteristics could be added to the questionnaire, while increasing the required overall intensity.

### 2.8 HS-SPME/GC–MS identification of volatile compounds

The volatile fragrant compounds in SFROs were identified according to the method by Xu's method (Xu et al., 2017). The SFROs were mixed with 200 μL of the internal standard solution which was dissolved into 0.4 mg/mL 4-octanol using methanol (Sigma Chemical Co., St. Louis, MO, USA) and preheated for 20 min at 80 °C. The volatile components were adsorbed on DVB/CAR/PDMS fibers (50 μm/30 μm coating, 1 cm length; Supelco, Bellefonte, PA, USA) at 80 °C for 20 min, then desorbed at 250 °C on a Shimadzu GC-TQ8040 gas chromatograph (Shimadzu, Kyoto, Japan) for 3 min. The Agilent DB-17MS column (60 m × 0.25 mm × 0.25 μm, split ratio of 50:1, 0.20 μm film thickness; Agilent Technologies, Wilmington, USA) was used, with helium as the carrier gas at a flow rate of 1.0 mL/min. The column temperature was set as follows: 40 °C for 3 min, then increased at a rate of 4 °C/min to 230 °C and held for 8 min. The qualitative identification of volatile compounds was assigned with a mass spectrum more than 85 % similarity to the MS NIST14 library (NIST14, version 2.2, National Institute of Standards and Technology, Gaithersburg, MD, USA), and quantitatively analyzed by internal standard method.

### 2.9 Statistical analysis

The experiment results were presented as mean ± standard deviation. The experimental group's differences were analyzed using One-way ANOVA with Duncan's multiple comparison post-hoc test with SPSS 20.0 and Origin Pro 9.0. The significant differences were considered at *P* < 0.05. Origin Pro 9.0 was used to plot the changing curves and figures.

## 3 Result and discussion

### 3.1 Physical/chemical properties of commercial peptide samples

The commercial rapeseed, soybean, corn and peanut peptides showed a light yellow, yellow, pale yellow and gray-white powder appearance, respectively. Using the methods of AOAC (1990, 15th), the contents of moisture, crude protein, residual sugar and crude ash in commercial rapeseed, soybean, corn and peanut peptides were determined and summarized in Table 1. The moisture contents of four commercial peptides ranged from 3.25 % to 4.36 %. Among them, peanut peptide had the highest moisture content of 4.36 %, and corn peptide had the lowest moisture content of 3.25 %, which mainly were due to their production/drying process and storage conditions. The rapeseed peptide contained the highest protein content of 885.2 g/kg, while peanut peptide had the lowest protein contents of 500.96 g/kg. Wang et al. determined that the soluble protein content in corn-soybean complex peptide prepared by hydrolysis method was 36.83 mg/mL (Wang et al., 2021). These four commercial peptides could be regarded to contain no reducing sugars with the contents of <0.10 g/kg.

As shown in Table 1, the rapeseed, soybean and corn peptides had the high percentages of the molecular weight range of 180–500 Da of 56.40 %, 53.72 % and 62.16 %, respectively, while peanut peptide with the molecular weight of >10,000 Da had the highest percentage of 67.92 %. The four commercial peptide samples contained 30.54 % (rapeseed peptide), 24.44 % (corn peptide), 16.76 % (peanut peptide) and 15.94 % (soybean peptide) of free amino acids (<180 Da). Further determination results of free amino acid compositions showed that total free amino acid contents in four commercial peptides ranged from 304.7 mg/kg to 884.7 mg/kg. Rapeseed peptide had the highest total free amino acid content (884.7 mg/kg), followed by soybean peptide (864.5 mg/kg), corn peptide (515.7 mg/kg) and peanut peptide (304.7 mg/kg). Among these free amino acids, glutamic acid showed the highest contents ranging from 112.8 to 202.8 mg/kg in four peptides, followed by leucine from 25.4 mg/kg to 130.9 mg/kg, which would provide the contribution to enhance the flavor of rapeseed oils as the substrates of Maillard reaction.

### 3.2 Effect of commercial peptides on the physicochemical properties of SRROs

After roasting of rapeseeds with 1 % (*w*/w) of glucose, 0.2 % (w/w) of xylose and 1–3 % of each commercial peptide, pressing at 80 °C, and degummed with 3 % (*v*/v) of hot water and centrifuging at 6000 ×g for 15 min, 12 strong rapeseed oils produced from rapeseed, soybean, corn and peanut peptides were named as R-SFROs (1 %, 2 % and 3 % of rapeseed peptide), S-SFROs (1 %, 2 % and 3 % of soybean peptide), C-SFROs (1 %, 2 % and 3 % of corn peptide) and P-SFROs (1 %, 2 % and 3 % of peanut peptide), respectively.

The acid value (AV) and peroxide value (PV), usually used as the important indicators for quality and refining (Zhang et al., 2024), were presented in Table 2. The acid values of four prepared SFROs ranged from 0.63 mg/g to 0.74 mg/g. Among them, the control sample had the lowest AV of 0.63 mg/g and P-SFRO (3 % of peanut peptide) had the highest content of 0.74 mg/g. Generally, the peroxide value mainly comes from oxidation process during storage. On this basis, the peroxide values of the fresh pressed R-SFROs, S-SFROs, C-SFROs and P-SFROs had no significant difference with the range of 1.50–1.62 mmol/kg (*P* > 0.05).

**Table 2:** Physicochemical results of SFROs produced by adding sugars and commercial peptides.

**Peptide types**	**Ratio (%)**	**AV (mg/g)**	**PV (mmol/kg)**	**Moisture and volatile matter**	**Lovibond Color**		
					**R**	**Y**	**W**
Control	/	0.63 ± 0.02^i^	1.58 ± 0.06^a^	0.125 ± 0.003^a^	3.8 ± 0.20^i^	35.0 ± 0.00^a^	0.2 ± 0.06^f^
Rapeseed peptide	1.0	0.7 ± 0.02^c^	1.56 ± 0.04^a^	0.115 ± 0.003^a^	4.6 ± 0.10^d^	35.0 ± 0.00^a^	0.5 ± 0.1^a^
	2.0	0.68 ± 0.01^e^	1.52 ± 0.01^a^	0.094 ± 0.002^a^	5.5 ± 0.50^b^	35.0 ± 0.00^a^	0.2 ± 0.12^e^
	3.0	0.72 ± 0.05^b^	1.53 ± 0.05^a^	0.124 ± 0.002^a^	6.5 ± 0.70^a^	35.0 ± 0.00^a^	0.3 ± 0.06^c^
Soybean peptide	1.0	0.66 ± 0.02^h^	1.5 ± 0.03^a^	0.112 ± 0.004^a^	4.3 ± 0.40^f^	35.0 ± 0.00^a^	0.4 ± 0.08^b^
	2.0	0.69 ± 0.04^d^	1.55 ± 0.05^a^	0.092 ± 0.001^a^	4.8 ± 0.20^c^	35.0 ± 0.00^a^	0.2 ± 0.04^e^
	3.0	0.7 ± 0.03^c^	1.62 ± 0.02^a^	0.087 ± 0.002^a^	5.4 ± 0.10^b^	35.0 ± 0.00^a^	0.1 ± 0.07^g^
Corn peptide	1.0	0.66 ± 0.01^h^	1.59 ± 0.03^a^	0.127 ± 0.002^a^	3.9 ± 0.20^h^	35.0 ± 0.00^a^	0.2 ± 0.06^f^
	2.0	0.71 ± 0.05^c^	1.61 ± 0.06^a^	0.119 ± 0.001^a^	4.5 ± 0.40^e^	35.0 ± 0.00^a^	0.3 ± 0.04^d^
	3.0	0.72 ± 0.07^b^	1.6 ± 0.02^a^	0.083 ± 0.002^a^	4.9 ± 0.10^c^	35.0 ± 0.00^a^	0.1 ± 0.03^h^
Peanut peptide	1.0	0.68 ± 0.04^e^	1.58 ± 0.01^a^	0.099 ± 0.002^a^	3.8 ± 0.50^i^	35.0 ± 0.00^a^	0.2 ± 0.07^e^
	2.0	0.72 ± 0.02^b^	1.53 ± 0.04^a^	0.102 ± 0.000^a^	4.1 ± 0.10^g^	35.0 ± 0.00^a^	0.3 ± 0.12^d^
	3.0	0.74 ± 0.01^a^	1.57 ± 0.03^a^	0.928 ± 0.001^a^	4.3 ± 0.50^f^	35.0 ± 0.00^a^	0.1 ± 0.09^i^

Note: Different letters indicate significant differences (P < 0.05). All experiments were performed in triplicates. The data were expressed as means ± standard deviations (n = 3).

Excessive moisture content accelerates the oxidation process during storage of vegetable oils (Zheng et al., 2018). Hang et al. found that increase of roasting temperature had a significant influence on the moisture and volatile matter content (Huang et al., 2024). In the present study, the roasting temperature and time were set as 160 °C and 20 min, respectively, which gave the stable moisture and volatile matter contents (<0.10 %) of 12 strong rapeseed oils, meeting the regulations in the Chinese National Standard GB/T 1536–2021 (Rapeseed oil) (≤0.15 %).

The color is a typical factor for judging or distinguishing the strong, mellow and light fragrant rapeseed oils (Tan et al., 2022). Generally, the pigments, chlorophyll and/or carotenoids and brown substances such as melanoidins and 5-hydroxymethylfurfural produced from Maillard reaction during roasting result in the dark color/appearance of edible oils (Carrín & Carelli, 2010; Zhang et al., 2024). The Lovibond color values of R-SFROs, S-SFROs, C-SFROs and P-SFROs were determined when setting the yellow value as 35.0. As shown in Table 2, the control sample showed the lowest red value of 3.8. Addition of commercial rapeseed, corn, soybean and peanut peptides significantly increased the red values of produced SFROs from 3.8 to 6.5 (*P* < 0.05). Among them, R-SFRO (3 % rapeseed peptide) had the highest red value of 6.5, which were attributed to the high contents of low-molecular-weight peptides (<500 Da) and high intensity of Maillard reaction. Zhang et al. also found that roasting conditions significantly influenced the color of rapeseeds and rapeseed oils due to the polymer pigments such as melanoidin originated from the Maillard reaction (Zhang, Fu, et al., 2023). Our previous work also indicated that the neutral protease and amylase-treatment led to the increase of Lovibond red reading to the maximum level of 6.0 (Tan et al., 2024).

### 3.3 Effect of commercial peptides on fatty acid profiles of SFROs

Previous studied had proved that the rapeseed oil is rich in unsaturated fatty acids (USFAs) occupying over 90 % of total fatty acids with the percentages of oleic acid (C18:1), followed by linoleic (C18:2) and α-linolenic acid (C18:3) ranging from 57 % to 64 %, 17 % to 22 %, and 6 % to 9 %, respectively (Tan et al., 2022).The roasting and refinery conditions seems to have little effect on the percentages of unsaturated fatty acids (USFAs) (Tan et al., 2022; Tan et al., 2023). Similar to those obtained conclusions, supplementation of sugars and commercial peptides showed no significant changes on the percentages of saturated fatty acids (SFAs) of 6.78–7.15 %, USFAs of 91.07–92.69 %, and MUSFAs (monounsaturated fatty acids) of 65.64–67.41 % (Table 3). The highest fatty acid was oleic acid (C18:1) with a stable level of approximate 47 %, followed by linoleic acid (C18:2) of 15.13–16.78 % and linolenic acid (C18:3) of 9.00 %, indicating all the produced SFROs should have a high nutritional value. Intestinally, the control sample contained higher percentage of trans-fatty acids (2.15 %) compared to those adding the sugars and commercial peptides (0.23–0.50 %). Similarly, Shen et al. found that the majority of rapeseeds contain over 70 % oleic acid and USFAs occupied approximately 90 % of the total fatty acid composition (Shen et al., 2023). Perrier et al. and Lewinska et al. also revealed that MUFAs account for the largest proportion of over 60 % of the total fatty acids in the rapeseed oil (Lewinska et al., 2015; Perrier et al., 2017).

**Table 3:** Fatty acid compositions and their relative percentages of SFROs produced by adding sugars and commercial peptides (%).

Items	Control	R-SFRO;1 %	R-SFRO;2 %	R-SFRO;3 %	S-SFRO;1 %	S-SFRO;2 %	S-SFRO;3 %	C-SFRO;1 %	C-SFRO;2 %	C-SFRO;3 %	P-SFRO;1 %	P-SFRO;2 %	P-SFRO;3 %
C14:0	0.04 ± 0.01^a^	0.04 ± 0.01^a^	0.05 ± 0.01^b^	0.04 ± 0.01^a^	0.05 ± 0.00^b^	0.05 ± 0.01^b^	0.04 ± 0.00^a^	0.04 ± 0.00^a^	0.04 ± 0.01^a^	0.04 ± 0.01^a^	0.05 ± 0.01^b^	0.05 ± 0.01^b^	0.04 ± 0.00^a^
C16:0	3.87 ± 0.04^c^	3.78 ± 0.04^e^	3.83 ± 0.06^d^	3.71 ± 0.08^g^	3.77 ± 0.05^f^	3.88 ± 0.02^c^	3.79 ± 0.05^e^	3.76 ± 0.05^f^	3.8 ± 0.00^e^	3.77 ± 0.03^f^	3.88 ± 0.05^c^	3.98 ± 0.08^a^	3.96 ± 0.05^b^
C16:1	0.18 ± 0.02^b^	0.19 ± 0.02^a^	0.20 ± 0.01^a^	0.20 ± 0.02^a^	0.20 ± 0.02^b^	0.20 ± 0.01^a^	0.20 ± 0.02^a^	0.19 ± 0.02^a^	0.19 ± 0.02^a^	0.19 ± 0.01^a^	0.20 ± 0.02^a^	0.20 ± 0.01^a^	0.20 ± 0.02^a^
C17:0	0.06 ± 0.02^a^	0.00	0.00	0.03 ± 0.00^b^	0.00	0.00	0.04 ± 0.01^b^	0.04 ± 0.01^b^	0.04 ± 0.02^b^	0.04 ± 0.01^b^	0.04 ± 0.01^b^	0.03 ± 0.01^b^	0.03 ± 0.01^b^
C17:1	0.06 ± 0.02^a^	0.05 ± 0.02^a^	0.06 ± 0.02^a^	0.06 ± 0.02^a^	0.06 ± 0.02^a^	0.06 ± 0.02^a^	0.06 ± 0.02^a^	0.06 ± 0.02^a^	0.06 ± 0.02^a^	0.06 ± 0.02^a^	0.06 ± 0.02^a^	0.07 ± 0.02^a^	0.07 ± 0.02^a^
C18:0	1.96 ± 0.03^b^	1.99 ± 0.03^a^	1.97 ± 0.02^b^	1.98 ± 0.03^a^	1.97 ± 0.01^b^	1.94 ± 0.03^b^	1.96 ± 0.01^b^	1.96 ± 0.03^b^	1.95 ± 0.02^c^	1.96 ± 0.03^b^	1.95 ± 0.01^c^	1.98 ± 0.03^a^	1.97 ± 0.03^b^
C18:1 t	0.11 ± 0.01^e^	0.30 ± 0.01^a^	0.32 ± 0.02^a^	0.28 ± 0.01^b^	0.30 ± 0.01^a^	0.30 ± 0.01^a^	0.28 ± 0.02^b^	0.27 ± 0.01^b^	0.29 ± 0.00^b^	0.26 ± 0.01^c^	0.24 ± 0.01^d^	0.30 ± 0.01^a^	0.13 ± 0.01^e^
C18:1	47.52 ± 0.87^b^	47.36 ± 0.81^d^	47.3 ± 0.4^e^	47.95 ± 0.35^a^	46.96 ± 0.41^g^	47.25 ± 0.82^f^	47.38 ± 0.54^d^	47.35 ± 0.33^d^	47.38 ± 0.51^d^	47.43 ± 0.46^c^	47.26 ± 0.75^e^	47.36 ± 0.87^d^	47.24 ± 0.31^f^
C18:2 t	0.03 ± 0.01^d^	0.07 ± 0.01b	0.09 ± 0.01^a^	0.08 ± 0.01^a^	0.09 ± 0.01^a^	0.08 ± 0.01^a^	0.08 ± 0.01^a^	0.08 ± 0.02^a^	0.09 ± 0.01^a^	0.07 ± 0.02^b^	0.06 ± 0.01^c^	0.09 ± 0.01^a^	0.03 ± 0.01^d^
C18:2	15.13 ± 0.05^e^	15.46 ± 0.08d	15.59 ± 0.11^c^	15.46 ± 0.03^d^	15.52 ± 0.06^c^	16.00 ± 0.12^b^	15.6 ± 0.08^c^	15.52 ± 0.08^c^	15.63 ± 0.05^c^	15.48 ± 0.06^d^	16.02 ± 0.08^b^	15.48 ± 0.18^d^	16.78 ± 0.02^a^
C18:3 t	1.01 ± 0.01^a^	0.08 ± 0.01b	0.09 ± 0.01^b^	0.07 ± 0.01^c^	0.08 ± 0.01^b^	0.07 ± 0.01^c^	0.07 ± 0.01^c^	0.07 ± 0.01^c^	0.08 ± 0.01	0.06 ± 0.01^c^	0.06 ± 0.01^c^	0.08 ± 0.01^b^	0.07 ± 0.01^c^
C18:3	9.33 ± 0.31^e^	9.47 ± 0.12c	9.42 ± 0.24^d^	9.05 ± 0.32^f^	9.47 ± 0.22^c^	9.37 ± 0.25^e^	9.46 ± 0.21^c^	9.42 ± 0.32^d^	9.36 ± 0.26^e^	9.40 ± 0.21^d^	9.56 ± 0.14^b^	9.67 ± 0.23^a^	9.23 ± 0.15^f^
C20:0	0.52 ± 0.01^d^	0.82 ± 0.01a	0.82 ± 0.02^a^	0.80 ± 0.01^b^	0.82 ± 0.02^a^	0.79 ± 0.01^b^	0.80 ± 0.01^b^	0.81 ± 0.02^a^	0.81 ± 0.02^a^	0.80 ± 0.01^b^	0.78 ± 0.01^c^	0.53 ± 0.00^d^	0.82 ± 0.02^a^
C20:1	8.44 ± 0.23^c^	8.29 ± 0.13f	8.25 ± 0.24^g^	8.3 ± 0.19^e^	8.36 ± 0.13^d^	8.79 ± 0.22^b^	8.21 ± 0.32^g^	8.24 ± 0.14^g^	8.18 ± 0.34^h^	8.27 ± 0.36^f^	8.77 ± 0.31^b^	8.86 ± 0.12^a^	8.13 ± 0.22^h^
C20:2	0.05 ± 0.02^d^	0.24 ± 0.02b	0.24 ± 0.01^b^	0.24 ± 0.02^b^	0.24 ± 0.00^b^	0.23 ± 0.02^c^	0.24 ± 0.01^b^	0.24 ± 0.02^b^	0.24 ± 0.02^b^	0.24 ± 0.02^b^	0.23 ± 0.02^c^	0.28 ± 0.01^a^	0.25 ± 0.02^b^
C22:0	0.22 ± 0.08^c^	0.31 ± 0.02a	0.31 ± 0.04^a^	0.31 ± 0.02^a^	0.31 ± 0.01^a^	0.30 ± 0.03^a^	0.30 ± 0.07^a^	0.31 ± 0.08^a^	0.31 ± 0.02^a^	0.31 ± 0.06^a^	0.3 ± 0.08^a^	0.31 ± 0.02^a^	0.31 ± 0.05^a^
C22:1	10.20 ± 0.24^d^	11.00 ± 0.11b	10.93 ± 0.24^c^	10.90 ± 0.14^c^	11.28 ± 0.11^a^	10.16 ± 0.13^e^	10.94 ± 0.26^c^	11.07 ± 0.08^b^	11.01 ± 0.16^b^	11.08 ± 0.21^b^	10.20 ± 0.34^e^	10.55 ± 0.26^d^	10.53 ± 0.28^d^
C22:2	0.02 ± 0.00^b^	0.08 ± 0.02a	0.08 ± 0.02^a^	0.08 ± 0.01^a^	0.08 ± 0.02^a^	0.07 ± 0.00^a^	0.08 ± 0.02^a^	0.08 ± 0.02^a^	0.08 ± 0.02^a^	0.08 ± 0.01^a^	0.07 ± 0.02^a^	0.00	0.00
C24:0	0.11 ± 0.03^c^	0.14 ± 0.01a	0.14 ± 0.03^a^	0.13 ± 0.01^b^	0.13 ± 0.01^b^	0.13 ± 0.02^b^	0.15 ± 0.04^a^	0.14 ± 0.01^a^	0.14 ± 0.03^a^	0.14 ± 0.03^a^	0.15 ± 0.02^a^	0.08 ± 0.01^d^	0.07 ± 0.01^d^
C24:1	0.14 ± 0.02^c^	0.33 ± 0.01b	0.33 ± 0.01^b^	0.33 ± 0.03^b^	0.33 ± 0.01^b^	0.32 ± 0.02^b^	0.33 ± 0.02^a^	0.36 ± 0.02^a^	0.34 ± 0.01^a^	0.34 ± 0.02^a^	0.32 ± 0.01^b^	0.10 ± 0.02^d^	0.14 ± 0.01^c^
SFA	6.78 ± 0.32^g^	7.08 ± 0.22c	7.12 ± 0.13^b^	7.00 ± 0.21^e^	7.05 ± 0.23^d^	7.09 ± 0.15^c^	7.08 ± 0.31^c^	7.06 ± 0.24^d^	7.09 ± 0.10^c^	7.06 ± 0.23^d^	7.15 ± 0.08^b^	6.96 ± 0.12^f^	7.2 ± 0.24^a^
USFA	91.07 ± 1.64^f^	92.47 ± 1.28^d^	92.40 ± 1.61^e^	92.57 ± 0.95^b^	92.50 ± 1.08^c^	92.45 ± 1.67^d^	92.50 ± 1.36^c^	92.53 ± 1.58^c^	92.47 ± 1.25^d^	92.57 ± 1.31^a^	92.69 ± 1.46^a^	92.57 ± 1.39^b^	92.57 ± 1.25^b^
PUSFA	25.43 ± 0.36^d^	25.25 ± 0.36^g^	25.28 ± 0.39^f^	25.29 ± 0.42^f^	25.34 ± 0.29^e^	25.62 ± 0.32^c^	25.26 ± 0.36^g^	25.22 ± 0.31^h^	25.27 ± 0.38^g^	25.16 ± 0.34^i^	25.83 ± 0.40^a^	25.65 ± 0.37^b^	25.65 ± 0.39^b^
TFA	2.15 ± 0.08^a^	0.45 ± 0.08c	0.50 ± 0.02^b^	0.43 ± 0.07^d^	0.47 ± 0.03^b^	0.45 ± 0.08^c^	0.43 ± 0.06^d^	0.42 ± 0.04^d^	0.46 ± 0.09^c^	0.39 ± 0.05^e^	0.36 ± 0.04^e^	0.47 ± 0.08^c^	0.23 ± 0.02^f^

Note: C14:0(myristic acid); C16:0(palmitic acid); C16:1(palmitoleic acid); C17:0(margaric acid); C17:1(cis-10-heptadecenoicacid); C18:0(stearic acid); C18:1(oleic acid); C18:2(linoleic acid); C18:3(linolenic Acid acid); C20:0(arachidic acid); C20:1(cis-11-eicosenoic acid); C20:2(11c,14c-eicosadienoic acid); C22:0(behenic acid); C22:1(erucic acid); C22:2(cis-13,16-docosadienoic acid); C24:0(lignoceric acid); C24:1(nervonic acid); SFA(saturated fatty acid); USFA(Unsaturated fatty acid); MUFA(Monounsaturated fatty acid); PUFA(Polyunsaturated fatty acid); TFA(Trans fatty acid). Different letters indicate significant differences (*P* < 0.05). All experiments were performed in triplicates. The data were expressed as means ± standard deviations (n = 3).

### 3.4 Effect of commercial peptides on nutrient contents of SFROs

**Fig. 1:**
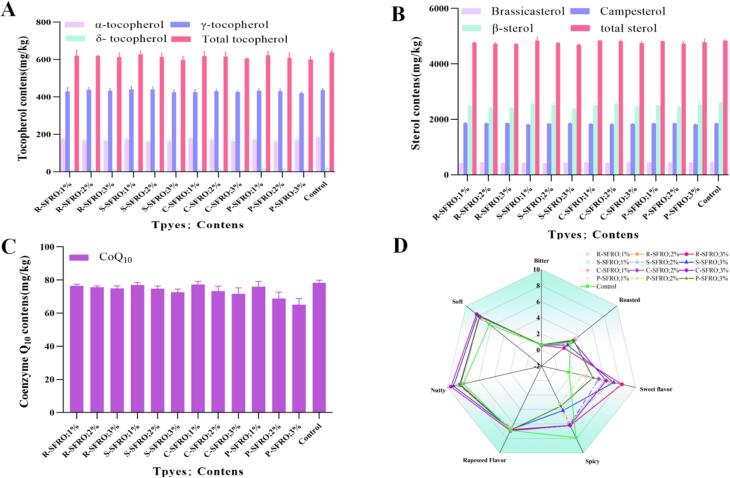
Nutrient contents in SFROs are produced by adding sugars and commercial peptides. (A), (B) and (C) represented the contents of tocopherols, sterol and CoQ_10_ in SFROs from untreated and different commercial peptides treated rapeseeds; **(D)** represented the aroma, spicy, bitter, rapeseed, sweet, nutty, soft and roasted flavors of SFROs produced by adding sugars and commercial peptides. Scores are based on a 10-point hedonic scale (0−10), with 0 representing the lowest score (not intense) and 10 representing the highest score (very intense). R-SFRO;1 %,R-SFRO;2 %,R-SFRO;3 %,S-SFRO;1 %,S-SFRO;2 %,S-SFRO;3 %,C-SFRO;1 %,C-SFRO;2 %,C-SFRO;3 %, P-SFRO;1 %, P-SFRO;2 %, P-SFRO;3 %, and Control represented the rapeseed peptide content in seeds treated by 1 % (rapeseed/1 %), rapeseed peptide content in seeds treated by 2 % (rapeseed/2 %), rapeseed peptide content in seeds treated by 3 % (rapeseed /3 %), seeds treated with 1 % soybean peptide content (soybean /1 %), seeds treated with 2 % soybean peptide content (soybean /2 %), seeds treated with 3 % soybean peptide content (soybean /3 %), seeds treated with 1 % corn peptide content (corn /1 %), seeds treated with 2 % corn peptide content (corn/1 %) Oil produced from seeds treated with 3 % corn peptide content (corn/3 %), seeds treated with 1 % peanut peptide content (peanut/1 %), seeds treated with 2 % peanut peptide content (peanut/2 %), seeds treated with 3 % peanut peptide content (peanut/3 %) and no commercial protein peptides were added, respectively.

Rapeseeds contain various fat-soluble nutrients including tocopherols, sterols, carotenoids and coenzyme Q_10_, which are co-extracted in rapeseed oils and proved to be beneficial for reducing the risk of degenerative nervous diseases (Shen et al., 2023). Fig. 1 presented the contents of total tocopherols, sterols and coenzyme Q_10_ in the produced SFROs. The contents of γ-tocopherol were the highest (420.17–440.58 mg/kg), followed by α-tocopherol (160.70–180.45 mg/kg) and δ-tocopherol (10.34–19.89 mg/kg), which were consistent with our previous work (Tan et al., 2022; Tan et al., 2024). Lewinska et al. found that tocopherol content was within the range of 8.3–727.6 mg/kg (Lewinska et al., 2015) and Zhang et al. also found that rapeseed oil contained up to 608.90 mg/kg of tocopherols (Zhang et al., 2022). The S-SFRO (3 % soybean peptide) contained the lowest content of the total tocopherol of 597.05 mg/kg, while the control sample had the highest total tocopherol level of 639.08 mg/kg, followed by S-SFRO (1 % soybean peptide) of 628.36 mg/kg. Similarly, Gao et al. also found that addition of Lys led to the highest total tocopherol content of 404.37 mg/kg with the α-, γ-, and δ-tocopherol contents of 183.06 mg/kg, 404.37 mg/kg and 12.69 mg/kg, respectively, compared to those in the groups added Arg, Pro, and Glu (Gao et al., 2024).

Sterol has a positive effect on regulating blood cholesterol content and preventing cardiovascular diseases (Kmiecik et al., 2020). As shown in Fig. 1 B, the total contents of sterols in the produced SFROs showed a similar trend to those of tocopherols, ranging from 4689.74 mg/kg to 4844.65 mg/kg. The control sample had the highest sterol content of 4844.65 mg/kg, with β-sitosterol, campesterol, and brassicasterol of 2382.95–2588.20 mg/kg, 1817.79–1869.47 mg/kg, and 418.16–451.59 mg/kg, respectively. Similarly, Roszkowska et al. found that the commercial rapeseed oils contained the total sterols contents ranging from 1999 mg/kg to 7122 mg/kg with β-sitosterol of 846–3388 mg/kg, campesterol of 762–2761 mg/kg and brassicasterol of 264–898 mg/kg, respectively (Roszkowska et al., 2015). Our previous results also showed that the neutral protease and amylase-treated rapeseeds gave the highest total sterol content of 4523.46 mg/kg (Tan et al., 2024).

Coenzyme Q_10_ is the third largest nutritional supplement after fish oil and vitamins and shows a significant potency to treat various diseases due to its powerful antioxidant activity and key physiological role in mitochondrial bioenergy (Arenas-Jal et al., 2020). As shown in Fig. 1 C, the coenzyme Q_10_ contents had no significant difference between the produced SFRO samples. For example, the coenzyme Q_10_ content in control sample was 78.41 mg/kg, while other SFROs produced by adding sugar and commercial peptides contained coenzyme Q_10_ with the range of 65.24–78.41 mg/kg. The content of coenzyme Q_10_ in rapeseed oil maintained at levels below 100 mg/kg, as mentioned in Fine et al's reference (Fine et al., 2016).

### 3.5 Sensory profile of SFROs produced by adding sugars and commercial peptides

Sensory evaluation grew rapidly along with the expansion of the processed food and consumer products industries. Using the accurate measurement of responses to develop foods, the consumer perception about the sensory characteristics of target products could be summarized from the collected useful information (Lawless & Heymann, 2010). As for the rapeseed oil products, the typical sensory properties, such as rapeseed flavor, burnt flavor, umami flavor, and bitterness are the key characteristics to distinguish their types and qualities (Zhang et al., 2024). In the present study, a 16-assessor panelist composed of research postgraduates from Jiangsu University, researchers and sales from Jiangsu Hefeng Grain and Oil Industry, Co., Ltd., and their customs were selected and trained to differentiate the flavors of prepared SFROs including bitter, roasted, sweet flavor, musty, spicy, rapeseed flavor, nutty and soft. A 10-point scale from “not intense” to “very intense” was set to record the intensity of each attribute. As shown in Table S1 and Fig. 1D, all the SFRO samples showed no musty or earthy flavor, and low bitter and spicy taste. Addition of commercial peptides and reducing sugars significantly improved the sweet, nutty and softy flavors of prepared SFROs to 1.86–8.26, 8.10–9.55 and 7.23–8.33, compared to those of control sample, respectively. The increase of peptide percentage also resulted in the enhancement of nutty and soft taste. In most cases, the nutty and soft flavors are regarded to be from the heterocyclic compounds including furans, pyrazines and pyrrole produced by the Maillard reaction (Starowicz & Zieliński, 2019). Herein, the increase of the nutty and soft flavors should be attributed to the reaction of added commercial peptides and reducing sugars. Among them, R-SFRO (3 % rapeseed peptide) showed the highest sweet, nutty and softy flavors of 8.26, 9.55 and 8.33, respectively, indicating that the rapeseed peptide containing higher percentages of amino groups (amino acids, lower-molecular-weight peptides) could react with reducing sugars to increase the intensity of Maillard reaction. Generally, rapeseeds contain a certain number of sulfur-compounds (glucosinolates, GSLs) (Prieto et al., 2019). Thermo-treatment degrades the GSLs to the glucosinolate degradation products (GDPs) such as 3-methyl-2-butenenitrile and 3-butyl isothiocyanate offering the spicy taste of rapeseed oils (Zhang et al., 2024). In the present study, the addition of 1 % of commercial peptides decreased the number of rapeseeds, which slightly decreased the spicy score to 6.32, compared to that of control sample (7.89).

### 3.6 Volatile compounds of SFROs with different commercial peptides

The SFRO's flavor is closely related to its quality, consumers' preference and marketing targets. From the published literature, over 300 volatile compounds including pyrazines, furans, nitriles sulfur (*S*)-containing compounds, and nitriles have been identified in the crude or commercial SFROs (Zhang et al., 2024). In the present study, a total of 108 volatile compounds were identified using GC/MS, including glucoside degradation products, pyrazines, furans, pyridines, thiophenes, thiazoles, aldehydes, ketones, acids, esters, alcohols, aromatic compounds and alkenes (Table S2).

**Fig. 2:**
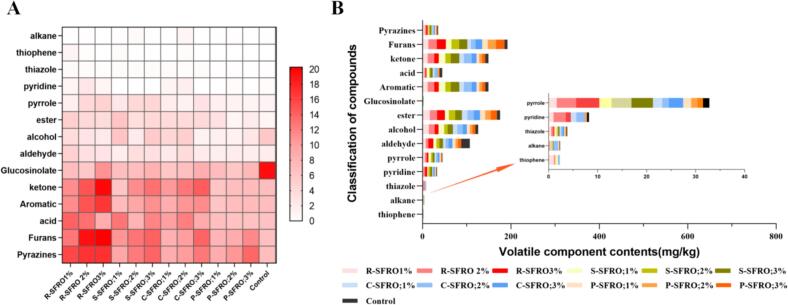
(A) Heat graphic of volatile components of rapeseed SFROs treated with different commercial peptides. The darker the red color, the higher the content of the corresponding volatile compound. (B) Stack charts of 13 SFROs volatile compounds. R-SFRO;1 %,R-SFRO;2 %,R-SFRO;3 %,S-SFRO;1 %,S-SFRO;2 %,S-SFRO;3 %,C-SFRO;1 %,C-SFRO;2 %,C-SFRO;3 %, P-SFRO;1 %, P-SFRO;2 %, P-SFRO;3 %, and Control represented the rapeseed peptide content in seeds treated by 1 % (rapeseed/1 %), rapeseed peptide content in seeds treated by 2 % (rapeseed/2 %), rapeseed peptide content in seeds treated by 3 % (rapeseed/3 %), seeds treated with 1 % soybean peptide content (soybean/1 %), seeds treated with 2 % soybean peptide content (soybean/2 %), seeds treated with 3 % soybean peptide content (soybean/3 %), seeds treated with 1 % corn peptide content (corn/1 %), seeds treated with 2 % corn peptide content (corn/1 %) Oil produced from seeds treated with 3 % corn peptide content (corn/3 %), seeds treated with 1 % peanut peptide content (peanut/1 %), seeds treated with 2 % peanut peptide content (peanut/2 %),seeds treated with 3 % peanut peptide content (peanut/3 %) and no commercial protein peptides were added, respectively. (For interpretation of the references to color in this figure legend, the reader is referred to the web version of this article.)

Glucosinolates including aliphatic, aromatic and indol types are the important secondary metabolite in cruciferous vegetables (Prieto et al., 2019). Generally, thioglycoside could be degraded by myrosinases, or high temperatures to produce the volatile compounds with spicy taste such as nitrile and isothiocyanat (Mao et al., 2019). On this basis, majority of the glucosinolates in rapeseeds was degraded during the roasting process, and finally contributing the spicy flavor of rapeseed oils. As shown in Fig. 2, the contents of glucoside degradation products (GDPs) ranged from 5.80 to 19.46 in all the tested SFROs. Among them, the control group had the highest GDPs content of 19.46 mg/kg, while other SFROs with the addition of 1–3 % commercial peptides showed a slightly decreased GDPs content of 5.80 to 11.01 mg/kg, which were in accordance with the trends of their sensory profiles. Among these glucoside degradation products, the content of Pentanenitrile, 5-(methylthio)- of 9.54 mg/kg was the highest, followed by Methallyl cyanide of 5.14 mg/kg and 1-Butene, 4-isothiocyanato- content of 1.90 mg/kg. Similarly, Bell et al. found that the temperature, pH, and enzymatic cofactors such as epithio specifier protein (ESP), thiocyanate formation protein (TFP) and nitrile specifier protein (NSP) significantly affected the contents of GDPs (Bell et al., 2018). Mao et al. found 60-min roasting at temperature of 150 °C decreased the total GSL contents of HZ, SC, QH, GS and DL rapeseeds by 30.47–84.44 % (Mao et al., 2019). Alcohol, ketones, acids, esters and aldehydes are common volatile substances formed by the oxidation of fats in vegetable oils, resulting in green, nutty, oily and even rotten smells (Mehretie et al., 2018). The formation of aldehydes occurs through lipid peroxidation and strecker degradation (Zhou et al., 2019). In the present study, the contents of acids and aldehyde in R-SFRO (1 % rapeseed peptide) were the highest content with 14.03 mg/kg and 4.86 mg/kg.

The reducing sugars and amino acids/peptide are the main substrates of the Maillard reaction, providing carbonyl, and amino groups to produce variety of heterocyclic compounds providing the unique food flavor. Generally, the types of amino compounds show various reaction rates. The amino acids and peptides have a higher reaction rate than that of proteins to produce the typical heterocyclic compounds such as pyrazines, furans, pyrroles, pyridines and thiophenes with a pleasant roasted and nut-like taste (Hang et al., 2021). For example, His, Lys, and Pro react with glucose to produce roasted nut flavor, while Gly, Ala, Tyr, and Asp with glucose produce caramel flavor (Liu et al., 2020). In the present study, the pyrazine content in the R-SFRO (3 % of rapeseed peptide) and S-SFRO (3 % of soybean peptide) were 17.17 mg/kg and 13.90 mg/kg, while control group contained 6.99 mg/kg of pyrazine. The pyrazine content in the R-SFRO (3 % of rapeseed peptide) was about 10 mg/kg higher than that in the control group, and it was found that the high pyrazine content increased with the increase of additional level of rapeseed peptide. The highest content of 2, 5-dimethylpyrazine was 7.06 mg/kg in R-SFRO. The furan contents in S-SFRO (3 % of rapeseed peptide) reached the highest levels of 20.26 mg/kg and pyrrole contents also reached 4.74 mg/kg, while that in the control group was 6.62 and 1.31 mg/kg, respectively, which also were in accordance with the sensory evaluation results. Similarly, Han et al. increased the supply of the reducing sugars (glucose, fructose, and maltose) and amino acids from flavored and alkaline protease-treated rapeseed meals and found that adding fructose enhanced the sweeter nutty/toasted flavor due to the formation of furfural and nitriles (Han et al., 2023). Tan et al. also found that the neutral protease and α-amylase-treatment of rapeseeds (NRO + ARO) increased the nutty and roasted fragrance and sweet flavor, mainly due to the highest contents of the formed pyrazines and furans originated from Maillard reactions (Tan et al., 2024).

### 3.7 Potency analysis of proposed method for large-scale application

Table 4 summarized and compared the process steps, energy input, and industrial potency of this work and from literature reports for enhancing the overall quality and volatile flavor of fragrant rapeseed oils. Generally, microwave, ultrasound and infrared roasting treatments of rapeseeds accelerate the processes of glucosinolate degradation, lipid thermal oxidation for increasing the volatile formation (Zhang et al., 2024). For example, Zhang et al. found that the 700-W microwave treatment of rapeseeds increased the heterocyclic compounds and glucosinolate degradation compounds (Zhang et al., 2023). Wang et al. pretreated the rapeseeds with infrared roasting (IR) method to increase the contents of nitriles/isothiocyanates and pyrazines offering the better flavors (Wang et al., 2023). Addition of other ingredients including herbs, spices, fruits, or vegetables also showed an improved effect on the odor attributes of rapeseed oils (Adams et al., 2011; Assami et al., 2016; Clodoveo et al., 2016). However, microwave, ultrasound and infrared roasting showed some limitations for industrial application due to the requirement of the expensive input of specific equipment, complex pretreating steps, and power consumption.

**Table 4:** Comparison between the literature results cited and this work for enhancing the flavors of SFROs.

**Methods**	**Operation conditions**	**Volatile flavor**	**Process Complexity**	**Energy consumption**	**Potency of large-scale application**	**References**
Rapeseed hull	Pretreatment: squeezing, grinding	The contents of dimethyl sulfide in “peeled” rapeseed oil was significantly higher than that in “unpeeled” rapeseed oil	++	+++	+++	Liu et al., 2018
Microwave treatment	700–800 W, 2450 MHz, 2-10 min heat 180 °C for 10 min	Woody, nutty, seed-like, baked, nutty, sweet, and almond-like odor notes. a transitional state from green and woody flavors to smoky, roasted and pungent flavors	+++	+++	++	Zhang et al., 2024; Zhang et al.,2023
Infrared roasting	Irradiation distance:80 mm roasting temperature:90–170 °C roasting time:30 min	More nitriles/isothiocyanates and pyrazines were generated Forming more pyrazines, isothiocyanates, nitrile and aldehydes	+++	+++	++	Yu et al., 2022; Wang et al.,2023
Flavoring oil	Pretreatment: add herbs, spices, fruits, vegetables	Form a small number of volatile components	+	+	+++	Adams et al., 2011; Asami et al., 2016; Clodoveo et al., 2016
Enzymatic treatment	Pretreatment: Rapeseed grinding, adding different proteases enzymatic hydrolysis. Pretreatment: rapeseed meal，amino acids, adjusted PH added protease, centrifuged enzymatic, added refined rapeseed oil mixed. Crushed rapeseed, added reducing sugar and amino acid, enzymatic hydrolysis, added refined rapeseed oil mixed.	Strong nutty, rich roasted fragrance and sweet flavor	++++	++++	+	Tan et al., 2024 Gao et al., 2024 Han et al.,2023
Addition of commercial peptides and reducing sugars	Directly add commercial peptides and reducing sugars with rapeseeds for roasting at 160 °C for 20 min	Low spicy flavor, strong nutty and rich sweet flavor	+	+	++++	This work

Note: The plus sign in the table indicates the degree from one to five and the degree from shallow to deep.

Increase of supplying the reducing sugars (glucose, fructose, and maltose) and amino acids by cellulose and/or α-amylase combined with protease treatment of rapeseeds or rapeseed meals also showed a significant increase of SFROs flavors (Gao et al., 2024; Han et al., 2023; Tan et al., 2024). For instance, Yuan et al. found the rapeseed oil having the significantly improvement of baking and caramel flavor with a high percentage of 2–5-dimethylpyrazine by co-heating the enzymatic hydrolysate of rapeseed cake with the refined rapeseed oil (Yuan et al., 2020). In most cases, enzymatic treatment would prolong the production process, increased the cost due to the high price of cellulose/α-amylase and proteases, and additionally the enzymatic-treated raw materials required the drying process. The present study proposed a method by direct addition of commercial rapeseed, soybean, corn or peanut peptide with residual sugars (glucose and xylose) to enhance the flavor intensity of SFROs with lower cost input (the price of commercial rapeseed, soybean, corn or peanut peptides of <50 CNY/kg) and no need of special pretreatment of rapeseeds.

## 4 Conclusions

The present study provided an economically and industrially applicable method to produce SFROs with the improved typical flavors by direct addition of commercial rapeseed, soybean, corn or peanut peptide with reducing sugars (glucose and xylose). Among them, R-SFRO (3 % rapeseed peptide) had the attractive color with highest red value of 6.5, showed a distinguishing strong nutty and a rich roasted fragrance and strong sweet flavor with highest overall score and contained the highest percentage of unsaturated fat acids (93.30 %), and the highest contents of Maillard reaction products such as pyrazines and furans. These findings provided an alternative method to produce the flavor-enhanced SFROs with high potency of industrial applications due to the simple steps and lower cost. The precise mechanisms of flavor enhancement and optimization for process parameters such as roasting temperature and time for the proposed method are ongoing in our lab for further industrial application.

## Funding

This work was supported by funding from Jiangsu Special Research and Development Grant for Northern Jiangsu Area, China (SZ-YC202145), and Science and Technology Plan Project of Dafeng District, Yancheng City (DFJH2023012).

## Compliance with ethical standards

### Ethical approval

Only voluntary adults were recruited to participate in the sensory evaluation such as appearance, and flavor characteristics of prepared SFROs. Informed, written consent was obtained from each participant in the study. Each of them could withdraw their consent without providing any justification. Each participant also consented to the processing of their personal data in accordance with the relevant guidelines and regulations included in ISO 8589-2007 (Sensory Analysis-General guidance for the design of test rooms) and GB/T 5525–2008 (Vegetable fats and oils-Method for identification of transparency, odor and flavor). Herein the ethical permission to conduct a human sensory study, is not a requirement of Jiangsu University.

## CRediT authorship contribution statement

**Zi-Xiong Zhou:** Writing – original draft, Methodology, Investigation. **Yu-Jun Chen:** Methodology, Data curation. **Ming-Ming Sheng:** Resources, Methodology. **Feng-Jie Cui:** Writing – original draft, Supervision, Resources, Project administration, Conceptualization. **Chen Chen:** Investigation, Data curation. **Jian-Cheng Shi:** Software, Resources. **Xue-Quan Shu:** Resources, Methodology. **Zhi-Wei Chen:** Supervision, Resources, Project administration.

## Declaration of competing interest

The authors declare that they have no known competing financial interests or personal relationships that could have appeared to influence the work reported in this paper.

## Data Availability

The datasets generated and/or analyzed during the current study are available from the corresponding author on reasonable request.

## Appendix A Supplementary data

Table S1 sensory profiles of SFROs produced by adding sugars and commercial peptides; Table S2 The contents and composition of volatile compounds of SFROs produced by adding sugars and commercial peptides. Image 1
