# Supplementary material for: Improving flavor of strong fragrant rapeseed oils by supplementing commercial peptides and sugars
Source: Food Chem X. 2024 Nov 8;24:101985. doi: 10.1016/j.fochx.2024.101985 (PMC11612813; doi:10.1016/j.fochx.2024.101985)
Supplement: Supplementary file 1 — Table S1 sensory profiles of SFROs produced by adding sugars and commercial peptides; Table S2 The contents and composition of volatile compounds of SFROs produced by adding sugars and commercial peptides. [file mmc1.docx]

**Improving Flavor of Strong Fragrant Rapeseed Oils by Supplementing Commercial Peptides and Sugars**

Zi-Xiong Zhou ^a^, Yu-Jun Chen ^b, c, d^, Ming-Ming Sheng^e^, Feng-Jie Cui ^a, *^, Chen Chen^a^, Jian-Cheng Shi ^b, d^, Xue-Quan Shu ^b, d^, Zhi-Wei Chen ^b, d^

*^a.^School of Food and Biological Engineering, Jiangsu University, Zhenjiang, 212013, P.R. China*

*^b.^ Jiangsu Hefeng Grain and Oil Industry Co., Ltd., Yancheng, 212002, P.R. China*

*^c^. Yancheng Hengxi Biotechnology Co., Ltd., Yancheng,* *224100, P.R. China*

*^d^. Jiangsu Jiafeng Grain and Oil Industry Co., Ltd., Yancheng,* *224100, P.R. China*

*^e^. Inspection and Testing Center of Dafeng, Yancheng, 224100, P.R. China*

** Corresponding Author:* Phone: +86-511-88780201; E-mail: [*fengjiecui@163.com*](mailto:fengjiecui@163.com)

**Table S1 sensory profiles of SFROs produced by adding sugars and commercial peptides**

| Evaluation | Bitter | Roasted | Sweet flavor | Musty | Earthy | Spicy | Rapeseed Flavor | Nutty | Soft |
| --- | --- | --- | --- | --- | --- | --- | --- | --- | --- |
| Control | 0.6±0.01^e^ | 2.71±0.34^h^ | 1.50±0.03^l^ | 0.00±0.00^a^ | 0.00±0.00^a^ | 7.89±0.56^a^ | 6.89±0.33^b^ | 7.96±0.16^i^ | 6.29±0.23^i^ |
| R-SFRO;1% | 0.6±0.03^e^ | 2.89±0.13^g^ | 5.62±0.85^f^ | 0.00±0.00^a^ | 0.00±0.00^a^ | 4.85±0.35^i^ | 6.68±0.11^j^ | 8.25±0.27^f^ | 7.23±0.25^h^ |
| R-SFRO;2% | 0.58±0.05^f^ | 2.56±0.2^j^ | 6.85±0.68^c^ | 0.00±0.00^a^ | 0.00±0.00^a^ | 5.86±0.25^g^ | 6.57±0.38^l^ | 8.65±0.33^b^ | 7.55±0.36^d^ |
| R-SFRO;3% | 0.55±0.02^h^ | 1.58±0.12^l^ | 8.26±1.50^a^ | 0.00±0.00^a^ | 0.00±0.00^a^ | 6.23±0.63^c^ | 6.93±0.27^a^ | 9.55±0.36^a^ | 8.33±0.41^a^ |
| S-SFRO;1% | 0.62±0.03^d^ | 3.25±0.23^d^ | 4.32±0.35^h^ | 0.00±0.00^a^ | 0.00±0.00^a^ | 6.12±0.32^d^ | 6.65±0.5^k^ | 8.16±0.58^g^ | 7.36±0.18^f^ |
| S-SFRO;2% | 0.59±0.02^f^ | 2.69±0.1^i^ | 6.12±0.60^de^ | 0.00±0.00^a^ | 0.00±0.00^a^ | 5.96±0.47^f^ | 6.69±0.33^i^ | 8.35±0.62^e^ | 7.65±0.33^d^ |
| S-SFRO;3% | 0.61±0.01^e^ | 2.16±0.23^k^ | 7.22±1.20^b^ | 0.00±0.00^a^ | 0.00±0.00^a^ | 4.15±0.52^j^ | 6.75±0.47^f^ | 9.23±0.47^a^ | 8.15±0.58^b^ |
| C-SFRO;1% | 0.68±0.05^a^ | 3.55±0.35^a^ | 3.65±0.25^i^ | 0.00±0.00^a^ | 0.00±0.00^a^ | 6.32±0.28^b^ | 6.73±0.26^g^ | 8.1±0.26^h^ | 7.35±0.47^f^ |
| C-SFRO;2% | 0.56±0.01^g^ | 3.32±0.4^c^ | 5.30±0.70^g^ | 0.00±0.00^a^ | 0.00±0.00^a^ | 6.12±0.26^d^ | 6.7±0.47^h^ | 8.52±0.47^c^ | 7.56±0.58^d^ |
| C-SFRO;3% | 0.63±0.03^c^ | 2.99±0.12^f^ | 6.25±0.35^d^ | 0.00±0.00^a^ | 0.00±0.00^a^ | 6.2±0.40^e^ | 6.8±0.58^e^ | 8.36±0.25^e^ | 7.84±0.62^c^ |
| P-SFRO;1% | 0.6±0.06^e^ | 3.5±0.23^b^ | 1.86±0.22^k^ | 0.00±0.00^a^ | 0.00±0.00^a^ | 5.45±0.63^h^ | 6.65±0.5^k^ | 8.35±0.53^e^ | 7.33±0.47^g^ |
| P-SFRO;2% | 0.56±0.02^g^ | 3.25±0.17^d^ | 3.21±0.15^ij^ | 0.00±0.00^a^ | 0.00±0.00^a^ | 3.23±0.14^l^ | 6.76±0.62^f^ | 8.65±0.25^b^ | 7.46±0.26^e^ |
| P-SFRO;3% | 0.65±0.04^b^ | 3.12±0.24^e^ | 4.56±0.48^h^ | 0.00±0.00^a^ | 0.00±0.00^a^ | 3.54±0.23^k^ | 6.85±0.36^c^ | 8.43±0.41^d^ | 7.85±0.41^c^ |

*Different letters indicate significant differences (*P* <0.05). All experiments were performed in triplicates. The data were expressed as means ± standard deviations (n=3).

**Table S2 The contents and composition of volatile compounds of SFROs produced by adding sugars and commercial peptides**

| **Classification** | **Name** | **CAS** | **Code**  **name** | **Contents(mg/kg)** | | | | | | | | | | | | |
| --- | --- | --- | --- | --- | --- | --- | --- | --- | --- | --- | --- | --- | --- | --- | --- | --- |
|  |  |  |  | **R-SFRO1%** | **R-SFRO 2%** | **R-SFRO3%** | **S-SFRO;1%** | **S-SFRO;2%** | **S-SFRO;3%** | **C-SFRO;1%** | **C-SFRO;2%** | **C-SFRO;3%** | **P-SFRO;1%** | **P-SFRO;2%** | **P-SFRO;3%** | **Control** |
| Glucosinolate  degradation  product | 2-Butenenitrile | 4786-20-3 | A1 | 1.13 | 1.26 | 0.99 | 0.83 | 0.88 | 0.56 | 0.28 | 0.85 | 0.96 | 0.83 | 0.88 | 0.56 | 0 |
|  | 3-Butenenitrile | 109-75-1 | A2 | 0.22 | 0.18 | 1.83 | 0 | 0 | 0 | 0 | 0 | 0 | 0 | 0 | 0 | 0 |
|  | 2,4-Pentadienenitrile | 1615-70-9 | A3 | 0 | 0.71 | 0 | 0 | 0 | 0 | 0 | 0 | 0 | 0 | 0 | 0 | 0 |
|  | 5-Cyano-1-pentene | 5048-19-1 | A4 | 0 | 1.43 | 1.32 | 0.74 | 0.4 | 0.7 | 0.4 | 0.37 | 0.39 | 0.54 | 0.1 | 0.3 | 2.5 |
|  | Pentanenitrile, 5-(methylthio)- | 59121-25-4 | A5 | 2.28 | 2.22 | 1.23 | 1.65 | 1.75 | 2.55 | 2.46 | 2.98 | 2.34 | 3.65 | 1.75 | 2.55 | 9.54 |
|  | Methylacrylonitrile | 126-98-7 | A6 | 0 | 0 | 0 | 1.26 | 0 | 0.72 | 1.13 | 1.11 | 1.49 | 0 | 0.26 | 0.53 | 0 |
|  | Benzenepropanenitrile | 645-59-0 | A7 | 0 | 0 | 2.05 | 0 | 1.95 | 2.03 | 0 | 0 | 1.52 | 0 | 1.53 | 1.96 | 0 |
|  | 6-(Methylthio)hexanenitrile | 72931-29-4 | A8 | 1.49 | 0 | 0.74 | 0 | 0 | 0 | 0 | 0 | 0 | 0 | 0 | 0 | 1.15 |
|  | Hexanenitrile, 5-methyl- | 19424-34-1 | A9 | 0 | 0 | 1.08 | 0.83 | 0 | 0 | 0 | 0 | 0 | 0.83 | 0 | 0 | 0 |
|  | 1-Cyano-4,5-epithiopentane | 58130-94-2 | A10 | 0 | 0 | 0 | 0 | 0 | 0 | 0 | 0.04 | 0 | 0 | 0 | 0 | 0 |
|  | Methallyl cyanide | 4786-19-0 | A11 | 0 | 0 | 0 | 0 | 0 | 0 | 0 | 0 | 0 | 0 | 0 | 0 | 5.14 |
|  | 1-Butene, 4-isothiocyanato- | 3386-97-8 | A12 | 1.9 | 0 | 1.77 | 1.63 | 1.85 | 1.55 | 1.77 | 1.39 | 1.76 | 1.23 | 1.55 | 1.62 | 1.13 |
|  | SUM |  |  | 7.02 | 5.80 | 11.01 | 6.93 | 6.83 | 8.11 | 6.04 | 6.74 | 8.46 | 7.08 | 6.07 | 7.52 | 19.46 |
| Pyrazines | Pyrazine, 2,5-dimethyl- | 123-32-0 | B1 | 6.28 | 6.59 | 7.06 | 5.92 | 5.06 | 6.51 | 3.02 | 3.07 | 6.51 | 2.33 | 2.23 | 3.63 | 1.97 |
|  | Pyrazine,2-ethyl-6-methyl- | 13925-3-6 | B2 | 0 | 0 | 0 | 0.62 | 0 | 0 | 0 | 0 | 0 | 0 | 0 | 0 | 0 |
|  | Pyrazine, trimethyl- | 14667-55-1 | B3 | 2.59 | 2.11 | 2.48 | 2.8 | 2.03 | 2.03 | 2.34 | 1.52 | 2.16 | 2.59 | 1.11 | 2.48 | 1.17 |
|  | Pyrazine, methyl- | 109-8-0 | B4 | 1.87 | 2.2 | 2.13 | 0 | 0 | 0 | 1.29 | 1.01 | 1.26 | 1.87 | 2.2 | 2.13 | 1.41 |
|  | Pyrazine, 2-ethyl-5-methyl- | 13360-64-0 | B5 | 1.07 | 0.76 | 1.04 | 0 | 1.63 | 1.63 | 0.87 | 1.07 | 0.88 | 1.07 | 0.78 | 1.04 | 0.6 |
|  | Pyrazine, 3-ethyl-2,5-dimethyl- | 13360-65-1 | B6 | 3.93 | 3.1 | 4.45 | 0 | 0 | 0 | 1.28 | 3.69 | 1.84 | 1.93 | 3.1 | 4.45 | 0 |
|  | Pyrazine, 2,6-diethyl- | 13067-27-1 | B7 | 0 | 0 | 0 | 0 | 3.73 | 3.73 | 0 | 0 | 0 | 0 | 0 | 0 | 0 |
|  | Pyrazine, 2,6-dimethyl- | 108-50-9 | B8 | 0 | 2.23 | 0 | 0 | 0 | 0 | 0 | 0 | 0 | 0 | 1.23 | 0 | 1.51 |
|  | 2-Acetyl-3-methylpyrazine | 23787-80-6 | B9 | 0 | 0 | 0 | 0 | 0 | 0 | 0 | 0 | 0 | 0 | 0 | 0 | 0.34 |
|  | SUM |  |  | 15.74 | 16.99 | 17.17 | 9.34 | 12.45 | 13.90 | 8.80 | 10.35 | 12.64 | 8.79 | 10.64 | 13.73 | 6.99 |
| Furans | Furan, 2-pentyl- | 3777-69-3 | C1 | 0 | 0 | 1.6 | 0 | 0 | 0 | 0 | 0 | 0 | 0 | 0 | 0 | 0.12 |
|  | Ethanone, 1-(2-furanyl)- | 1192-62-7 | C2 | 1.29 | 1.71 | 1.48 | 1.33 | 1.65 | 1.74 | 1 | 1.09 | 1.42 | 1.33 | 1.65 | 1.74 | 0.5 |
|  | 2-Furancarboxaldehyde, 5-methyl- | 620-2-0 | C3 | 4.2 | 5.97 | 5.54 | 2.09 | 2.11 | 3.8 | 2.57 | 3.28 | 4.01 | 1.09 | 2.11 | 3.8 | 1.98 |
|  | 3-Furanmethanol | 4412-91-3 | C4 | 3.95 | 6.5 | 5.97 | 3.98 | 4.05 | 3.8 | 2.42 | 3.1 | 4.83 | 1.98 | 2.05 | 3.25 | 1.99 |
|  | 2-Furanmethanol, 5-methyl- | 3857-25-8 | C5 | 0.2 | 0.32 | 0.31 | 0.28 | 0.32 | 0.42 | 0.23 | 0.2 | 0.26 | 0.28 | 0.32 | 0.42 | 0.17 |
|  | 2(5H)-Furanone | 497-23-4 | C6 | 1.04 | 1.31 | 1.51 | 0.89 | 1.25 | 1.38 | 0.69 | 0.83 | 1.07 | 0.89 | 1.25 | 0.38 | 0.49 |
|  | Furaneol | 3658-77-3 | C7 | 0 | 0.91 | 0.74 | 0.54 | 0.88 | 1.2 | 0 | 0 | 0.63 | 0.54 | 0.88 | 0.2 | 0 |
|  | Furyl hydroxymethyl ketone | 17678-19-2 | C8 | 1.88 | 2.33 | 2.37 | 1.48 | 2.13 | 1.43 | 1.26 | 1.57 | 1.63 | 1.48 | 0.13 | 1.43 | 0.64 |
|  | 5-Acetoxymethyl-2-furaldehyde | 10551-58-3 | C9 | 0 | 0 | 0.31 | 0 | 0 | 0 | 0 | 0 | 0 | 0 | 0 | 0 | 0 |
|  | 5-(Hydroxymethyl) dihydrofuran-2(3H)-one | 10374-51-3 | C10 | 0.09 | 0.16 | 0.34 | 0.13 | 0.13 | 0.18 | 0.13 | 0.1 | 0.09 | 0.13 | 0.13 | 0.18 | 0.06 |
|  | 2,5-Dimethyl-2,4-dihydroxy-3(2H)-thiophenone | 96504-28-8 | C11 | 0 | 0.08 | 0.09 | 0.07 | 0.05 | 0.18 | 0.04 | 0.04 | 0 | 0.07 | 0.05 | 0.18 | 0 |
|  | 3-Methyl-2-furoic acid | 4412-96-8 | C12 | 0 | 0 | 0 | 0 | 0.27 | 0 | 0 | 0 | 0 | 0 | 0.27 | 0 | 0 |
|  | 2,5-Dimethylfuran-3,4(2H,5H)-dione | 68755-49-7 | C13 | 0 | 0 | 0 | 0 | 0 | 0 | 0 | 0 | 0 | 0 | 0 | 0 | 0.34 |
|  | 3(2H)-Furanone, 4-hydroxy-5-methyl- | 19322-27-1 | C14 | 0 | 0 | 0 | 0 | 0 | 0 | 0 | 0 | 0 | 0 | 0 | 0 | 0.2 |
|  | Benzofuran, 2,3-dihydro- | 496-16-2 | C15 | 0 | 0 | 0 | 0 | 0 | 0 | 0 | 0 | 0 | 0 | 0 | 0 | 0.13 |
|  | Furan, 2-ethyl- | 3208-16-0 | C16 | 0 | 0 | 0 | 0 | 0 | 0 | 0 | 0 | 0 | 0 | 0 | 0 | 0 |
|  | SUM |  |  | 12.65 | 19.29 | 20.26 | 10.79 | 12.84 | 14.13 | 8.34 | 10.21 | 13.94 | 7.79 | 8.84 | 11.58 | 6.62 |
| pyrrole | 2-Pyrrolidinone, 1-methyl- | 872-50-4 | D1 | 0 | 0.77 | 0.86 | 0 | 0 | 0.8 | 0.66 | 0 | 0.69 | 0 | 0 | 0 | 0 |
|  | Ethanone, 1-(1H-pyrrol-2-yl)- | 1072-83-9 | D2 | 1.63 | 1.24 | 1.54 | 1.26 | 1.68 | 1.49 | 1.25 | 1.35 | 1.15 | 0.85 | 0.73 | 0.67 | 0.73 |
|  | 1H-Pyrrole-2-carboxaldehyde, 1-methyl- | 1192-58-1 | D3 | 0 | 1.94 | 2.34 | 1.26 | 2.38 | 2.08 | 0 | 0 | 1.05 | 0.8 | 0.58 | 0.45 | 0.58 |
|  | SUM |  |  | 1.63 | 3.95 | 4.74 | 2.52 | 4.06 | 4.37 | 1.91 | 1.35 | 2.89 | 1.65 | 1.31 | 1.12 | 1.31 |
| pyridine | Pyridine | 110-86-1 | E1 | 0.98 | 0 | 0.45 | 0 | 0 | 0 | 0 | 0 | 0 | 0 | 0.31 | 0 | 0.31 |
|  | 1,2-Ethanediol, 1,2-di-4-pyridinyl- | 1844571 | E2 | 0 | 2.52 | 0 | 0 | 0 | 0 | 1.15 | 1.68 | 0 | 0 | 0 | 0 | 0 |
|  | Pyridine, 2-methyl- | 109-6-8 | E3 | 0 | 0 | 0.54 | 0 | 0 | 0 | 0 | 0 | 0 | 0 | 0 | 0 | 0 |
|  | Ethanone, 1-(2-pyridinyl)- | 1122-62-9 | E4 | 0 | 0 | 0 | 0 | 0 | 0 | 0 | 0.09 | 0 | 0 | 0.1 | 0 | 0.1 |
|  | SUM |  |  | 0.98 | 2.52 | 0.99 | 0.00 | 0.00 | 0.00 | 1.15 | 1.77 | 0.00 | 0.00 | 0.41 | 0.00 | 0.41 |
| thiophene | Thiophene | 110-2-1 | F1 | 1.08 | 0 | 0 | 0 | 0 | 0 | 0 | 0.42 | 0 | 0 | 0 | 0 | 0 |
|  | 2-Thiophenemethanol | 636-72-6 | F2 | 0 | 0.27 | 0 | 0.51 | 0 | 0 | 0 | 0 | 0 | 0 | 0 | 0 | 0 |
|  | SUM |  |  | 1.08 | 0.27 | 0.00 | 0.51 | 0.00 | 0.00 | 0.00 | 0.42 | 0.00 | 0.00 | 0.00 | 0.00 | 0.00 |
| Thiazole | 5-Thiazoleethanol, 4-methyl- | 137-0-8 | G1 | 0.52 | 0.37 | 0.27 | 0.39 | 0.33 | 0.32 | 0.34 | 0.28 | 0.34 | 0.21 | 0.15 | 0.13 | 0.15 |
|  | SUM |  |  | 0.52 | 0.37 | 0.27 | 0.39 | 0.33 | 0.32 | 0.34 | 0.28 | 0.34 | 0.21 | 0.15 | 0.13 | 0.15 |
| alcohol | 1-Penten-3-ol | 616-25-1 | H1 | 0.43 | 0 | 0 | 0 | 0.24 | 0.48 | 0 | 0.49 | 0 | 0.32 | 0 | 0.14 | 0 |
|  | Methyl Alcohol | 67-56-1 | H2 | 0.09 | 0 | 0.34 | 0.3 | 0.17 | 0.05 | 0.1 | 0.12 | 0.06 | 0.09 | 0.05 | 0.05 | 0.05 |
|  | 1-Eicosanol | 629-96-9 | H3 | 0.83 | 0 | 0.56 | 0 | 0 | 0.85 | 0.73 | 0.79 | 0 | 0.38 | 0 | 0 | 0 |
|  | Maltol | 118-71-8 | H4 | 1.48 | 1.34 | 0.99 | 1.32 | 1 | 1.17 | 0.95 | 1.05 | 0.83 | 0.53 | 0.55 | 0.42 | 0.55 |
|  | 4H-Pyran-4-one, 3,5-dihydroxy-2-methyl- | 1073-96-7 | H5 | 0.71 | 0.51 | 0.36 | 0.66 | 0.57 | 0.43 | 0.4 | 0.35 | 0.49 | 0.15 | 0.25 | 0.26 | 0.25 |
|  | 1-Hexacosanol | 506-52-5 | H6 | 0 | 0.91 | 0 | 0 | 0 | 0 | 0 | 0 | 0 | 0 | 0 | 0 | 0 |
|  | 2-Penten-1-ol, (Z)- | 1576-95-0 | H7 | 0 | 0 | 0 | 3.96 | 0 | 2.64 | 2.04 | 2.41 | 0 | 0 | 0 | 0 | 0 |
|  | 2-Butanol, 3-methyl-, acetate | 5343-96-4 | H8 | 0 | 0 | 0 | 0 | 0.11 | 0 | 0 | 0 | 0 | 0 | 0 | 0 | 0 |
|  | Ethanol, 1,1'-oxybis-, diacetate | 10526-21-3 | H9 | 0 | 0 | 0 | 0 | 0 | 0 | 0.08 | 0 | 0 | 0.03 | 0 | 0.07 | 0 |
|  | Phenylethyl Alcohol | 22258 | H10 | 0 | 0 | 0 | 0 | 0 | 0 | 0 | 0 | 0 | 0 | 0.59 | 0.68 | 0.59 |
|  | 1,5-Hexadien-3-ol | 924-41-4 | H11 | 0 | 0 | 0 | 0 | 0 | 0 | 0 | 0 | 0 | 0 | 0 | 0 | 4.41 |
|  | 2,3-Butanediol, [R-(R*,R*)]- | 24347-58-8 | H12 | 0 | 0 | 0 | 0 | 0 | 0 | 0 | 0 | 0 | 0 | 0 | 0.43 | 0 |
|  | Propylene Glycol | 57-55-6 | H13 | 0 | 0 | 0 | 0 | 0 | 0 | 0 | 0 | 0 | 0 | 0 | 0.48 | 0 |
|  | SUM |  |  | 3.54 | 2.76 | 2.25 | 6.24 | 2.09 | 5.62 | 4.3 | 5.21 | 1.38 | 1.5 | 1.44 | 2.53 | 5.85 |
|  | Hexanal | 66-25-1 | I1 | 0.86 | 0 | 0.52 | 0 | 0.48 | 0.56 | 0.48 | 0.58 | 0.41 | 0.38 | 0.31 | 0.32 | 0.31 |
| aldehyde | Acetaldehyde, hydroxy- | 141-46-8 | I2 | 0.15 | 0 | 0 | 0 | 0 | 0 | 0 | 0 | 0 | 0 | 0 | 0 | 0 |
|  | Nonanal | 124-19-6 | I3 | 1.29 | 0.19 | 0 | 0 | 0.26 | 0 | 0 | 0.23 | 0 | 0 | 0.18 | 0.16 | 0.18 |
|  | 2,4-Heptadienal, (E,E)- | 881395 | I5 | 0.86 | 1.04 | 0.88 | 0.6 | 0.98 | 0.96 | 0.73 | 1.1 | 0.6 | 0.47 | 0.45 | 0.37 | 0.45 |
|  | 2-Decenal, (E)- | 3913-81-3 | I6 | 0.62 | 0.7 | 0.56 | 0.33 | 0.59 | 0.72 | 0.5 | 0.61 | 0.41 | 0.32 | 0 | 0 | 0 |
|  | Benzaldehyde, 4-hydroxy-3,5-dimethoxy- | 134-96-3 | I7 | 0.22 | 0 | 0 | 0.3 | 0.2 | 0.21 | 0 | 0.14 | 0 | 0.15 | 0.13 | 0.2 | 0.13 |
|  | 2,4-Heptadienal, (E,E)- | 881395 | I8 | 0.86 | 1.04 | 0.88 | 0.6 | 0.98 | 0.96 | 0.73 | 1.1 | 0.6 | 0.47 | 0.45 | 0.37 | 0.45 |
|  | Benzeneacetaldehyde | 122-78-1 | I9 | 0 | 0.27 | 0 | 0 | 0 | 0 | 0 | 0 | 0 | 0 | 0 | 0.03 | 0 |
|  | Benzaldehyde, 4-methyl- | 104-87-0 | I10 | 0 | 0 | 0 | 0 | 0.02 | 0 | 0 | 0 | 0.02 | 0 | 0 | 0 | 0 |
|  | Heptanal | 111-71-7 | I11 | 0 | 0 | 0 | 0 | 0 | 0 | 0 | 0 | 0 | 0 | 0.11 | 0 | 0.11 |
|  | Benzaldehyde | 100-52-7 | I12 | 0 | 0 | 0 | 0 | 0 | 0 | 0 | 0 | 0 | 0 | 0.31 | 0.33 | 0.31 |
|  | SUM |  |  | 4.86 | 3.24 | 2.84 | 1.83 | 3.51 | 3.41 | 2.44 | 3.76 | 2.04 | 1.79 | 1.94 | 1.78 | 1.94 |
|  | 2-Pentanone | 107-87-9 | J1 | 1.2 | 0.08 | 1.63 | 0.33 | 0.74 | 0.88 | 0.44 | 0.86 | 1.83 | 0.68 | 0.38 | 0.56 | 0.38 |
|  | 2,3-Pentanedione | 600-14-6 | J2 | 1.17 | 0.21 | 0.99 | 0 | 0.96 | 0.31 | 0.56 | 0.96 | 1.53 | 0.79 | 0.39 | 0.9 | 0.39 |
|  | 2-Propanone, 1-hydroxy- | 116-9-6 | J3 | 1.11 | 0.75 | 1.56 | 0.05 | 0.5 | 0.73 | 0.36 | 0.56 | 1.28 | 0.7 | 0.93 | 0.74 | 0.93 |
| ketone | 4-Cyclopentene-1,3-dione | 930-60-9 | J4 | 0.62 | 0.7 | 0.38 | 0.63 | 0.37 | 0.53 | 0.4 | 0.51 | 1.39 | 0.21 | 0 | 0 | 0 |
|  | 4H-Pyran-4-one, 2,3-dihydro-3,5-dihydroxy-6-methyl- | 28564-83-2 | J5 | 7.22 | 5.16 | 7.23 | 3.56 | 8.35 | 8.36 | 8.83 | 8.72 | 8.38 | 3.88 | 5.14 | 4.96 | 5.14 |
|  | [1,3]Diazepan-2,4-dione | 75548-99-1 | J6 | 0 | 8 | 7.60 | 1.59 | 0 | 2.28 | 0 | 1.13 | 0 | 0 | 0 | 0 | 0 |
|  | Ethanone, 1-(4-hydroxy-3,5-dimethoxyphenyl)- | 2478-38-8 | J7 | 0 | 0 | 0 | 0.12 | 0 | 0 | 0 | 0 | 0 | 0 | 0 | 0 | 0 |
|  | Gallacetophenone-4'-methylether | 708-53-2 | J8 | 0 | 0 | 0 | 0 | 0.46 | 0 | 0 | 0 | 0 | 0 | 0.16 | 0 | 0.16 |
|  | (S)-(+)-4-Isopropyl-2-oxazolidinone | 17016-83-0 | J9 | 0 | 0 | 0.25 | 0 | 0 | 0 | 0 | 0 | 0 | 0 | 0 | 0.11 | 0 |
|  | SUM |  |  | 11.32 | 14.9 | 19.79 | 6.28 | 11.38 | 13.09 | 10.59 | 12.74 | 14.41 | 6.26 | 7 | 7.27 | 7 |
|  | Acetic acid | 64-19-7 | K1 | 5.32 | 3.8 | 3.59 | 4.81 | 2.48 | 3.23 | 2.4 | 2.87 | 2.08 | 1.92 | 3.13 | 3.01 | 3.13 |
|  | Butanoic acid, 4-hydroxy- | 591-81-1 | K2 | 2.68 | 2.78 | 0 | 3.55 | 0 | 2.88 | 1.91 | 2.26 | 1.82 | 1.42 | 1.52 | 1.52 | 1.52 |
|  | 4-Pentenoic acid | 591-80-0 | K3 | 3.29 | 3.45 | 2.76 | 2.65 | 2.59 | 3.36 | 2.38 | 2.5 | 2.05 | 1.44 | 0.58 | 0.51 | 0.58 |
| acid | 5-Hexenoic acid | 1577-22-6 | K4 | 1.35 | 1.39 | 1.29 | 1.53 | 1.02 | 1.33 | 1.05 | 1.17 | 0.9 | 0.65 | 0 | 0 | 0 |
|  | Erucin | 4430-36-8 | K5 | 0.31 | 0.27 | 0.25 | 0 | 0.15 | 0.24 | 0.16 | 0.21 | 0.15 | 0.12 | 0 | 0 | 0 |
|  | Pentanoic acid, 4-oxo- | 123-76-2 | K6 | 0.83 | 0 | 0 | 0 | 0.52 | 0.59 | 0.48 | 0.37 | 0.41 | 0 | 0.15 | 0 | 0.15 |
|  | Benzoic acid | 65-85-0 | K7 | 0.25 | 0.35 | 0.23 | 0.33 | 0.37 | 0.24 | 0.24 | 0.12 | 0.24 | 0.18 | 0.07 | 0.13 | 0.07 |
|  | Propanoic acid, 2,2-dimethyl-, anhydride with diethylborinic acid | 34574-27-1 | K8 | 0 | 1.02 | 0 | 0 | 0 | 0 | 0 | 0 | 0 | 0 | 0 | 0 | 0 |
|  | Nonanoic acid | 112-5-0 | K9 | 0 | 0 | 0.18 | 0 | 0 | 0 | 0 | 0.23 | 0 | 0 | 0 | 0 | 0 |
|  | n-Hexadecanoic acid | 21096 | K10 | 0 | 0 | 0.27 | 0 | 0 | 0 | 0 | 0.28 | 0 | 0 | 0 | 0 | 0 |
|  | Hexanoic acid | 142-62-1 | K11 | 0 | 0 | 0 | 0 | 0.91 | 0 | 1.43 | 1.31 | 1.28 | 0.98 | 0.64 | 0.94 | 0.64 |
|  | Cyclotetrasiloxane, octamethyl- | 556-67-2 | K12 | 0 | 0 | 0 | 0 | 0 | 0 | 0 | 0.58 | 0 | 0 | 0 | 0 | 0 |
|  | Tetradecanoic acid | 544-63-8 | K13 | 0 | 0 | 0 | 0 | 0 | 0 | 0 | 0.35 | 0 | 0 | 0 | 0 | 0 |
|  | Pentanoic acid, 3-methyl- | 105-43-1 | K14 | 0 | 0 | 0 | 0 | 0 | 0 | 0 | 0 | 0 | 0 | 0.42 | 0.45 | 0.42 |
|  | SUM |  |  | 14.03 | 13.06 | 8.57 | 12.87 | 8.04 | 11.87 | 10.05 | 12.25 | 8.93 | 6.71 | 6.5 | 6.57 | 6.51 |
|  | Pivalic acid vinyl ester | 3377-92-2 | L1 | 1.42 | 0 | 1.02 | 1.14 | 1.02 | 1.17 | 0.99 | 1 | 1.2 | 0.85 | 0.61 | 0.7 | 0.61 |
|  | Butanoic acid, 2-methyl-3-oxo-, ethyl ester | 609-14-3 | L2 | 0 | 0.78 | 0 | 0.69 | 0 | 0 | 0 | 0 | 0 | 0.21 | 0 | 0 | 0 |
|  | Methyl 2-furoate | 611-13-2 | L3 | 0 | 0 | 0 | 0 | 0 | 0 | 0 | 0 | 0 | 0 | 0 | 0.43 | 0 |
| ester | 2,5-Furandione, dihydro-3-methyl- | 4100-80-5 | L4 | 0 | 0 | 0 | 0 | 0 | 0 | 0 | 0 | 0 | 0 | 0 | 0.66 | 0 |
|  | 2H-Pyran-2-one,tetrahydro1,2-Cyclopentanedione, | 823-22-3 | L5 | 3.51 | 3.26 | 2.67 | 4.36 | 2.48 | 2.93 | 2.82 | 2.78 | 2.72 | 2.06 | 0 | 0 | 0 |
|  | 3-methyl- | 765-70-8 | L6 | 0 | 0 | 0 | 0 | 0 | 0 | 0 | 0 | 0 | 0 | 0.4 | 0.27 | 0.4 |
|  | SUM |  |  | 4.93 | 4.04 | 3.68 | 6.19 | 3.50 | 4.11 | 3.81 | 3.78 | 3.92 | 3.12 | 1.01 | 2.06 | 1.01 |
|  | 2-Methoxy-4-vinylphenol | 7786-61-0 | M1 | 2.15 | 2.06 | 2.84 | 2 | 2.39 | 3.42 | 1.93 | 2.12 | 1.73 | 1.27 | 1.46 | 1.39 | 1.46 |
|  | Phenol, 4-ethenyl-2,6-dimethoxy- | 28343-22-8 | M2 | 7.91 | 7.12 | 7.84 | 5.28 | 6.99 | 7.67 | 5.7 | 5.16 | 6.44 | 4.99 | 4.95 | 5.2 | 4.95 |
|  | Phenylethyl Alcohol | 22258 | M3 | 1.26 | 0 | 6.12 | 0 | 0 | 0 | 0.96 | 3.46 | 3.24 | 0 | 0.59 | 0.68 | 0.59 |
| Aromatic compound | SUM |  |  | 11.32 | 14.9 | 16.79 | 7.28 | 9.38 | 11.09 | 8.59 | 10.74 | 11.41 | 6.26 | 7 | 7.27 | 7 |
|  | 1,5-Hexadiene, 2,4-dimethyl- | 68701-71-3 | N1 | 0 | 0 | 0 | 0.12 | 0.05 | 0 | 0 | 0 | 0 | 0 | 0 | 0 | 0 |
|  | n-Hexane | 110-54-3 | N2 | 0 | 0.18 | 0 | 0 | 0 | 0.09 | 0 | 0 | 0 | 0 | 0 | 0 | 0 |
|  | Undecane | 1120-21-4 | N3 | 0.11 | 0 | 0 | 0 | 0 | 0 | 0 | 0 | 0 | 0 | 0 | 0 | 0 |
| alkane | Acetonitrile | 27522 | N4 | 0 | 0 | 0 | 0 | 0.4 | 0 | 0 | 0 | 0 | 0.18 | 0 | 0 | 0 |
|  | 1,6-Bis(2-propyn-1-yloxy)hexane | 55720-49-5 | N5 | 0 | 0 | 0 | 0 | 0 | 0 | 0 | 0.98 | 0 | 0 | 0 | 0 | 0 |
|  | Oxirane, [(1-methylethoxy)methyl] | 4016-14-2 | N6 | 0 | 0 | 0 | 0 | 0 | 0 | 0 | 0 | 0 | 0 | 0.11 | 0 | 0.11 |
|  | SUM |  |  | 0.11 | 0.18 | 0 | 0.12 | 0.45 | 0.09 | 0 | 0.98 | 0 | 0.18 | 0.11 | 0 | 0.11 |
